# Wheat photosystem II heat tolerance responds dynamically to short- and long-term warming

**DOI:** 10.1093/jxb/erac039

**Published:** 2022-05-23

**Authors:** Bradley C Posch, Julia Hammer, Owen K Atkin, Helen Bramley, Yong-Ling Ruan, Richard Trethowan, Onoriode Coast

**Affiliations:** 1 ARC Centre of Excellence in Plant Energy Biology, Division of Plant Sciences, Research School of Biology, The Australian National University, Canberra, ACT 2601, Australia; 2 Department of Biology, The University of Western Ontario, 1151 Richmond St, N6A 3K7, London, Canada; 3 Plant Breeding Institute, Sydney Institute of Agriculture & School of Life and Environmental Sciences, The University of Sydney, Narrabri, NSW 2390, Australia; 4 Australia-China Research Centre for Crop Improvement and School of Environmental and Life Sciences, The University of Newcastle, Callaghan, NSW 2308, Australia; 5 School of Life and Environmental Sciences, Plant Breeding Institute, Sydney Institute of Agriculture, The University of Sydney, Cobbitty, NSW 2570, Australia; 6 Natural Resources Institute, University of Greenwich, Central Avenue, Chatham Maritime, Kent ME4 4TB, UK; 7 School of Environmental and Rural Sciences, University of New England, Armidale, NSW 2351, Australia

**Keywords:** Acclimation, chlorophyll fluorescence, heat stress, phenotypic plasticity, photosynthesis, photosystem II, thermotolerance, *Triticum* species

## Abstract

Wheat photosynthetic heat tolerance can be characterized using minimal chlorophyll fluorescence to quantify the critical temperature (*T*_crit_) above which incipient damage to the photosynthetic machinery occurs. We investigated intraspecies variation and plasticity of wheat *T*_crit_ under elevated temperature in field and controlled-environment experiments, and assessed whether intraspecies variation mirrored interspecific patterns of global heat tolerance. In the field, wheat *T*_crit_ varied diurnally—declining from noon through to sunrise—and increased with phenological development. Under controlled conditions, heat stress (36 °C) drove a rapid (within 2 h) rise in *T*_crit_ that peaked after 3–4 d. The peak in *T*_crit_ indicated an upper limit to PSII heat tolerance. A global dataset [comprising 183 *Triticum* and wild wheat (*Aegilops*) species] generated from the current study and a systematic literature review showed that wheat leaf *T*_crit_ varied by up to 20 °C (roughly two-thirds of reported global plant interspecies variation). However, unlike global patterns of interspecies *T*_crit_ variation that have been linked to latitude of genotype origin, intraspecific variation in wheat *T*_crit_ was unrelated to that. Overall, the observed genotypic variation and plasticity of wheat *T*_crit_ suggest that this trait could be useful in high-throughput phenotyping of wheat photosynthetic heat tolerance.

## Teaser

Heat tolerance of wheat PSII was highly plastic in response to changing growth conditions. An upper limit to increasing heat tolerance was identified following a short-term heat stress.

## Introduction

As the climate changes, global mean land surface temperature has continued to rise, alongside more frequent, longer, and more intense heatwaves (Perkins-Kirkpatrick and Lewis, 2020). This is particularly concerning for the prospect of improving crop yields, as heat stress is associated with significant declines in the yield of widely cultivated crops, including wheat (Asseng *et al.*, 2015; Tack *et al.*, 2015; Hochman *et al.*, 2017; Liu *et al.*, 2019; Ortiz-Bobea *et al.*, 2019). Photosynthesis is a primary determinant of wheat yield, and it is particularly sensitive to heat stress (Berry and Bjorkman, 1980; Way and Yamori, 2014). Improving the heat tolerance of photosynthesis could future-proof wheat yield in a warming world (Cossani and Reynolds, 2012; Scafaro and Atkin, 2016; Iqbal *et al.*, 2017). To realize improvements in wheat photosynthetic heat tolerance, it is paramount that we first understand and quantify patterns of wheat photosynthetic heat tolerance so that we can then successfully exploit them.

Decreased leaf photosynthetic rate under high temperature is partially linked to disruption of the chloroplast electron transport chain, of which the thylakoid membrane-embedded PSII is considered the most sensitive component (Sharkey, 2005; Brestic *et al.*, 2012). Heat-induced reactive oxygen species and lipid peroxidation both cause cleavage of the reaction centre-binding D1 protein of PSII (Yamashita *et al.*, 2008), inhibiting electron flow and thus the production of ATP. For decades PSII damage has been measured with Chl *a* fluorescence metrics, including the critical temperature of F_0_ (henceforth *T*_crit_) (Schreiber *et al.*, 1975; Schreiber and Berry, 1977; Hüve *et al.*, 2011; Geange *et al.*, 2021). *T*_crit_ is the critical temperature above which minimal Chl *a* fluorescence (F_0_) rises rapidly, indicating incipient damage to PSII (Schreiber and Berry, 1977; Melcarek and Brown, 1979; Neuner and Pramsohler, 2006; Slot *et al.*, 2019). *T*_crit_ is associated with increased thylakoid membrane fluidity, disruption of the light-harvesting antennae (Raison *et al.*, 1982; Figueroa *et al.*, 2003), dissociation of chloroplast membrane-bound proteins (Berry and Bjorkman, 1980), and loss of chloroplast thermostability (Armond *et al.*, 1978). As a standardized metric, *T*_crit_ has been used to examine global patterns of heat tolerance, quantify phenotypic plasticity in response to warming, and assess vulnerability to climate change across plant species (O’Sullivan *et al.*, 2017; Zhu *et al.*, 2018; Lancaster and Humphreys, 2020; Geange *et al.*, 2021). While the number of publications examining plant *T*_crit_ is growing (Ferguson *et al.*, 2020; Arnold *et al.*, 2021; Slot *et al.*, 2021), most studies focused on woody, non-crop species, and characterization of intraspecies variation in *T*_crit_ of crop species has been limited (see Ferguson *et al.*, 2020 for a recent exception). Wheat, as the most widely cultivated crop (with >220 Mha cultivated worldwide) with a diverse range of genotypes originating from across the globe, is an ideal crop species for examining intraspecies variation and acclimation of *T*_crit_. In addition, although wheat is a temperate crop, there is increasing evidence of warming in many wheat-producing regions, including China, the USA, and Australia, resulting in either stalled or reduced wheat yield (Hochman *et al.*, 2017; Zhao *et al.*, 2017). Understanding the response of *T*_crit_ to warming and the magnitude of intraspecies variation in *T*_crit_ could thus provide opportunities for improving photosynthetic heat tolerance and yield resilience in wheat and other crops.

Quantification of intraspecific variation in physiological traits of crops commonly encounters bottlenecks at the phenotyping stage. However, high-throughput phenotyping techniques are being developed, including a robotic system offering a 10-fold increase in the measurement speed of dark respiration (Scafaro *et al.*, 2017; Coast *et al*., 2019, 2021), and the proximal remote sensing of leaf hyperspectral reflectance signatures for rapidly assaying photosynthetic characteristics and dark respiration (Silva-Pérez *et al.*, 2018; Coast *et al.*, 2019; Fu *et al.*, 2019). Though chlorophyll fluorescence techniques are well established for assessing photosynthetic heat tolerance, they are typically cumbersome. This limits their incorporation in breeding programmes that screen hundreds of genotypes for heat tolerance. However, recently, Arnold *et al.* (2021) described a high-throughput chlorophyll fluorescence screening technique for a diversity of wild species. Previous studies of photosynthetic thermal tolerance also largely assumed that *T*_crit_ is diurnally and phenologically constant. However, these assumptions may be flawed. Substantial changes in metabolic capacity and demand for photosynthetic products vary diurnally, with phenological development and in response to fluctuations in temperature (Steer, 1973; Rashid *et al.*, 2020). Thus, it seems reasonable that *T*_crit_ may demonstrate similar variation. However, these assumptions remain untested.

The extent to which plants physiologically adjust to warming is important in determining productivity and survival (Scheiner, 1993; Leung *et al.*, 2020). Acclimation of photosynthetic electron transport to elevated temperature is evidenced by an increase in *T*_crit_. Zhu *et al.* (2018) reported acclimation at a rate of a 0.34 °C increase in *T*_crit_ for every 1 °C increase in average temperature over the growing season for a range of Australian species. Acclimation of *T*_crit_ may also increase plant thermal safety margins, thus protecting against damage to PSII under future heat stress. Thermal safety margins are estimated as the difference between the upper limit of leaf function (e.g. *T*_crit_) and the maximum growth temperature experienced in an environment (Sastry and Barua, 2017), and they provide a useful representation of a species’ potential vulnerability to global warming (Sunday *et al.*, 2014). A reduction in this margin indicates increasing vulnerability to heat stress, while an increase in this margin indicates better capacity to tolerate the effects of climate warming (Hoffmann *et al.*, 2013). Thermal safety margins of 10–15 °C have been reported for many plant species (Weng and Lai, 2005; O’Sullivan *et al.*, 2017; Perez and Feeley, 2020), with some as high as 12–31 °C (Leon-Garcia and Lasso, 2019) when leaf temperature, rather than air temperature, was used. However, many plant species have low thermal safety margins (e.g. ≤5 °C; Sastry and Barua, 2017). Unfortunately, reports quantifying the acclimation capacity and thermal safety margins of food crops are scarce. Reports on acclimation of *T*_crit_ to warming have been in response to a sustained increase in long-term growth temperature. Similar descriptions of *T*_crit_ acclimation to short-term heat stress (e.g. heatwaves) are not well documented. Considering that heatwaves are predicted to become more frequent and intense (Perkins-Kirkpatrick and Lewis, 2020), it is pertinent that we understand if and how *T*_crit_ responds to heatwaves. Whether acclimation of *T*_crit_ to heatwaves has an upper threshold (i.e. a ceiling temperature) is currently unknown.

Previous uses of *T*_crit_ to assess global patterns of heat tolerance have been underpinned by ecological theories established in terrestrial ectotherms and endotherms (Addo-Bediako *et al.*, 2000; Deutsch *et al.*, 2008; Sunday *et al.*, 2011; Araújo *et al.*, 2013). One such theory is that organism physiology correlates closely with large-scale geographical patterns in the thermal environment where populations of an individual species evolved (Gabriel and Lynch, 1992). Indeed, greater photosynthetic heat tolerance of non-crop plants originating from hotter, equatorial environments has been reported for numerous species (O’Sullivan *et al.*, 2017; Drake *et al.*, 2018; Zhu *et al.*, 2018; Lancaster and Humphreys, 2020). It remains unknown whether such global patterns of interspecies variation hold for intraspecific comparisons, for example in a widely cultivated crop such as wheat, with genotypes originating from across the globe.

In this study, we employed a high-throughput system to describe intraspecies variation and high temperature acclimation of *T*_crit_ in wheat. Our objectives were to: (i) examine whether leaf *T*_crit_ varies diurnally and with phenology; (ii) determine the thermal safety margins and assess vulnerability of wheat to high temperatures in the Australian grain belt; and (iii) to assess if there is an upper threshold for leaf *T*_crit_ exposed to a sustained heat shock. To achieve these objectives, we conducted three field studies and one controlled-environment experiment. In addition, we conducted a systematic literature review of wheat *T*_crit_ and used the global data we generated to investigate if intraspecies variation in wheat leaf *T*_crit_ is related to the latitude of genotype (as a proxy for climate of origin) of wheat genotypes or species.

## Materials and methods

### Field experiments: assessing diel and phenological variation in wheat *T*_crit_ and estimating thermal safety margins of Australian wheat

#### Germplasm

A set of 20–24 wheat genotypes (Supplementary Table S1) were used in three field experiments conducted in Australia across 3 years. Twenty of these genotypes were used by Coast *et al.* (2021) to assess acclimation of wheat photosynthesis and respiration to warming in two of the fields. The genotypes included: commercial Australian cultivars; heat-tolerant materials developed by the centres of the Consultative Group on International Agricultural Research (CGIAR) in Mexico and Morocco and tested in warm areas globally; materials derived from targeted crosses between adapted hexaploid cultivars and heat-tolerant Mexican hexaploid landraces; tetraploid emmer wheat (*T*. *dicoccon* Schrank ex Schübl.), Indian cultivars; and synthetic wheat derived by crossing *Aegilops tauchii* with modern tetraploid durum wheat. All genotypes evaluated were hexaploid and chosen for their contrasting heat tolerance under high temperature conditions in Sudan (Gezira; 14.9°N, 33°E), Australia (Narrabri, NSW; 30.27°S, 149.81°E), and Mexico (Ciudad Obregón; 27.5°N, 109.90°W).

#### Experimental design and husbandry

The first 2 years of field experiments were undertaken in regional Victoria (2017, Dingwall; and 2018, Barraport West), and the third was in regional New South Wales (2019, Narrabri). A detailed description of the experimental designs for the 2017 and 2018 experiments is given in Coast *et al.* (2021). Briefly, a diverse panel of genotypes were sown on three dates each in 2017 (20 genotypes) and 2018 (24 genotypes) to expose crops to different growth temperatures at a common developmental stage. The first time of sowing (TOS) for both experiments was within the locally recommended periods for sowing (early May). Subsequent sowing times were 1 month apart in June and July. Experiments were sown in three adjacent strips, one for each TOS. Each strip consisted of four replicate blocks. The 2019 field experiment was similar in all aspects to the 2018 field experiment, except for the following: (i) only two sowing times were incorporated in the design; and (ii) the sowing times were ~2 months apart (17 May 2019 and 15 July 2019). Of the 24 genotypes sown in 2018, only 20, which were common to the 2017 and 2019 experiments, were assessed for *T*_crit_. All three field experiments were managed following standard agronomic practices for the region by the Birchip Cropping Group (www.bcg.org.au) in regional Victoria, and staff of the IA Watson Grains Research Centre at The University of Sydney, and Australian Grain Technologies, in Narrabri. A summary of the field experiments is presented in Table 1.

**Table 1.** Information on field experiments in Australia

Experiment location and year	Year	Genotypes studied ^*a*^	Mean daily maximum temperature at anthesis (°C)	Radiation (μmol photons m ^-2 ^ s ^-1 ^)^*b*^
Dingwall, Victoria	2017	20	21.4–31.6	1394–1934
Barraport West, Victoria	2018	20	22.8–33.4	1706–2331
Narrabri, New South Wales	2019	24	22.6–34.1	1823–1950
**Experiment objective**	**TOS** ^*c*^	**Genotypes studied**	**Brief description of method**	
Diurnal variation in *T*_crit_				
Dingwall, Victoria	3	6	Flag leaf *T*_crit_ determined at anthesis at four consecutive time points occurring every 6 h over an 18 h period (i.e. solar noon, sunset, midnight, and sunrise).	
Phenological variation in *T*_crit_				
Barraport West, Victoria	1–3	4	Flag leaf *T*_crit_ determined at heading, anthesis, and grain filling on the same day at 10.00 h from all three time of sowing plots.	
Rate of acclimation of *T*_crit_^*d*^ and ­calculation of thermal safety margins				
Dingwall, Victoria	1–3	20	Times of sowing varied so that plants sown later experienced warmer growth environments at a common developmental stage. Flag leaf *T*_crit_ determined at anthesis. Thermal safety margins were estimated as the difference between genotype mean flag leaf *T*_crit_ at anthesis and the maximum recorded air temperature at Dingwall/Barraport West (40 **°**C) or Narrabri (40.8 **°**C) in October (typical month of peak wheat anthesis).	
Barraport West, Victoria	1–3	20		
Narrabri, New South Wales	1–2	24		

^*a*^ Twenty genotypes were common to all experiments. The designation of all genotypes used in this study is provided in Supplementary Table S1.

^*b*^ Mean maximum photosynthetically active radiation measured with Licor 6400XTs light sensors.

^*c*^ Time of sowing, where the first time of sowing was within the locally recommended sowing window, with subsequent times of sowing separated in 1 month intervals at Victoria, or 2 months at New South Wales.

^*d*^ An additional *T*_crit_ high temperature acclimation study was conducted under controlled environments with two of the 24 genotypes.

#### Diel measurements of wheat *T*_crit_

Six of the 20 genotypes in the 2017 field experiment at Dingwall, Victoria were used to investigate diel variation. The six genotypes were two commercial cultivars (Mace and Suntop) and four breeding lines (with reference numbers 143, 2254, 2267, and 2316). These were chosen because they are representative of the diversity of the set of 20 genotypes (Coast *et al.*, 2021). To determine if *T*_crit_ varied diurnally, one flag leaf from each of four replicate plants was harvested at anthesis [Zadok growth stage (GS) 60–69; Zadoks *et al.*, 1974] from plants of TOS 3 at four consecutive time points occurring every 6 h over an 18 h period (solar noon, sunset, midnight, and sunrise).

#### Phenological measurements of wheat *T*_crit_

A subset of four genotypes from the 20 in the 2018 field experiment at Barraport West, Victoria was used to assess phenological variation in *T*_crit_. The four genotypes were the breeding lines 2062, 2150, 2254, and 2267, the latter two of which were also used for diel measurements as described in the previous paragraph. Plants at heading (Zadok GS50–59), anthesis (Zadok GS60–69), and grain filling (Zadok GS70–79) were respectively chosen from fields of the three sowing times. One flag leaf was harvested from the tallest tiller of each replicate plant (minimum eight replicates) at the different phenological stages at 10.00 h on the same day and used to determine *T*_crit_.

#### Estimation of thermal safety margin of Australian wheat

All 20 genotypes in the 2017 and 2018 field experiments in Dingwall and Barraport West respectively, as well as all 24 genotypes in the 2019 field experiment in Narrabri were used to estimate thermal safety margins. Thermal safety margins were estimated as the difference between individual genotype *T*_crit_ and the maximum recorded air temperature at either Dingwall or Narrabri in October. Similar definitions of thermal safety margins as the difference between the measured temperature at which a species experiences irreversible physiological damage and the maximum measured temperature of the species’ habitat have been used in studies of animal ectotherms and plants (Deutsch *et al.*, 2008; Sunday *et al.*, 2014; O’Sullivan *et al.*, 2017; Sastry and Barua, 2017). We considered Barraport West and Dingwall together for their historical weather records as they are in close proximity to one another in the Mallee district of Victoria, Australia. Weather data for Dingwall and Barraport West were obtained from the Australian Bureau of Meteorology covering the period 1910–2020. We used 40 °C and 40.8 °C (the maximum recorded air temperature for October) in Dingwall/Barraport West and Narrabri, respectively, to quantify thermal safety margins under current climatic scenarios. October was chosen as the upper threshold of exposure of field plants at anthesis to heat because all the later sown plants in this study were at anthesis in October. For future climatic conditions, we added 2.6 °C and 5 °C to the current maximum temperature, with the 2.6 °C addition representing the top end of the intermediate emission Representative Concentration Pathway (RCP) 4.5 IPCC scenario predicted for Eastern Australia by 2090 (1.3–2.6 °C), and the 5 °C addition similarly representing the top end of the high emission RCP 8.5 IPCC scenario predicted for Eastern Australia by 2090 (2.8–5 °C; Climate Change Australia, 2021). Across all times of sowing in the three field experiments, flag leaves were harvested at anthesis (Zadok GS60–69) at a standardized time of 09.00–10.000 h to determine *T*_crit_ and estimate thermal safety margins.

### Controlled-environment experiment: speed of acclimation and upper limit of leaf *T*_crit_ during heat shock

A controlled-environment experiment was conducted to determine the speed and threshold of the response of *T*_crit_ to a sudden heat shock. Two wheat genotypes, 29 and 2267 (Supplementary Table S1), which contrasted in *T*_crit_ under common conditions were used to assess the speed and potential threshold of the response of wheat leaf *T*_crit_ to sudden heat shock. This experiment was conducted at the Controlled Environment Facilities of the Australian National University (ANU), Canberra, Australia.

#### Plant husbandry

Seeds were germinated on saturated paper towels in covered plastic containers under darkness for 1 week. Germinated seedlings were planted in 1.05 litre pots (130 mm diameter) filled with potting mix (80% composted bark, 10% sharp sand, 10% coir) with 4g l^–1^ fertilizer (Osmocote Exact Mini fertiliser, ICL, Tel Aviv, Israel) mixed through.

#### Temperature treatment

Potted plants were grown in glasshouses in which a 24/18 °C day/night temperature regime with a 12 h photo-thermal period was maintained until tillering. At tillering, when all plants had a fully extended third leaf (Zadok GS22–29; Zadoks *et al.*, 1974), plants were moved into growth cabinets (TPG-2400-TH, Thermoline Scientific, Wetherill Park, NSW, Australia) for temperature treatment. One of two temperature conditions were imposed: a day/night regime of 24/18 °C, or a heat shock with day/night temperatures of 36/24 °C. White fluorescent tubes provided a 12 h photoperiod of photosynthetically active radiation of 720–750 µmol m^−2^ s^−1^ at plant height. Leaf discs were sampled from fully extended third leaves from main tillers and used to determine *T*_crit_ after 2, 4, 24, 48, 72, and 120 h in the growth cabinets. Four replicate plants were used for *T*_crit_ measurement at each sampling time and for each temperature condition. Plant husbandry followed standard practice at the ANU Controlled Environment Facilities.

### Meta-study (field experiments, glasshouse studies, and a systematic literature review) of wheat *T*_crit_ relationship with origins of genotypes

To explore how our results compare with previous studies that have assessed wheat leaf *T*_crit_, and whether genotypes from hot habitats exhibit higher *T*_crit_, we undertook a systematic review of the published literature and compiled data from >30 years (1988–2020) of wheat leaf *T*_crit_ studies. A database was generated using information from a recently published systematic review on global plant thermal tolerance (Geange *et al.*, 2021) and additional literature search. These published data were combined with data from the three field experiments described above. The multiple times of sowings in each of the three Australian field experiments provided us with eight thermal environments for obtaining wheat leaf *T*_crit_ from a total of 24 wheat genotypes. We also included unpublished wheat leaf *T*_crit_ data from nine other experiments conducted in controlled-environment facilities at the Australian National University. Overall, our global dataset included 3223 leaf *T*_crit_ samples from 183 wheat genotypes of various species (*Triticum aestivum* L., *T. turgidum* L., ssp. durum Desf., *T. turgidum* L., ssp. diococcoides Thell.), and wild wheat (*Aegilops* species).

### Determination of leaf *T*_crit_

One leaf per plant was harvested and stored in plastic bags alongside a saturated paper towel and were left to dark adapt for a minimum of 20 min while being transported back to the laboratory. There was ~60–90 min between harvest and measurement time, which was maintained consistently across all experiments. Previous trial experiments with the fluorometer demonstrated that change in wheat *T*_crit_ post-harvest is minimal (< 0.5 °C) within this time frame. Water (90 μl) was placed in each well of a 48-well Peltier heating block in order to ensure that leaf samples remained hydrated throughout the assay. A single 6 mm diameter leaf disc was excised from the middle of each harvested dark-adapted leaf and placed within each well of the heating block. Once discs were all loaded into the heating block, a glass plate was used to enclose the wells to prevent leaf pieces from drying out during the assay. The block was then placed directly beneath the lens of an imaging fluorometer (FluorCam 800MF, Photon Systems Instruments, Brno, Czech Republic) and programmed to heat from 20 °C to 65 °C at a rate of 1 °C min^−1^. Prior independent tests using copper–constantan thermocouples [42 SWG (standard wire gauge), 64 μm diameter] referenced against a PT-100 platinum resistance thermometer confirmed that changes in programmed block temperature were strongly correlated with leaf temperature in all wells. The fluorometer recorded F_0_ throughout the heating period (approximately one record per minute). Following the conclusion of the temperature ramp, fluorescence data were processed and used to estimate *T*_crit_, which was calculated according to the method described by Schreiber and Berry (1977), using the R package ‘segmented’ (Muggeo, 2008). Briefly, the package identifies the breakpoint in data containing a broken-line relationship by estimating linear and generalised linear models. This breakpoint in the F_0_ curve was taken as *T*_crit_.

### Statistical analysis

Statistical analyses were carried out within the R statistical environment (v. 3.4.4; R Core Team, 2018) with R Studio. For analysis of the field data on diurnal and phenological variation in *T*_crit_, we employed linear mixed models in R using the packages lmerTest (Kuznetsova *et al.*, 2017) and emmeans (Lenth, 2020). Genotype was a fixed term in all models, while other fixed terms were time of day (for analysis of diel variation), developmental stage (for analysis of phenological variation), and time of sowing (for analysis of growth temperature variation), with replicate included as a random term in all models. The ceiling threshold of *T*_crit_ under heat stress (of 36 °C), in the controlled-environment experiment, was determined by fitting a non-linear regression to the *T*_crit_ by time relationship. Then using the coefficients of the fitted regressions, we estimated the time at which the fitted *T*_crit_ was highest, and this was taken as the time to peak acclimation. To test the relationships between *T*_crit_ and growth environment temperature, we only used data from the three field experiments in Australia, for which we had reliable data. The 24 genotypes studied under field conditions in Australia were grouped based on the region of origin of their pedigree (Aleppo, Syria; Gezira, Sudan; Narrabri, Australia; Obregón, Mexico; Pune, India; and Roseworthy, Australia) and the relationship was examined using linear or bivariate regressions. Our global dataset (see Table 2 for sources) was used to ascertain the link between wheat leaf *T*_crit_ and climate of origin by regressing mean genotype *T*_crit_ with genotype latitude (as a proxy for climate) of origin.

**Table 2.** Source of data used for assessment of global variation in leaf photosynthetic heat tolerance (*T*_crit_)

Study ^*a*^	Origin	Species	Mean *T*_crit_ (*n*)
This study	Asia	*Triticum aestivum* L.	45.1 (8)
	Africa	*T. aestivum* L.	44.6 (1)
	Australia	*T. aestivum* L. and *T. dicoccum* Schrank	44.7 (9)
	North America	*T. aestivum* L.	45.0 (6)
**Average**			**44.8 (24)**
Havaux *et al.* (1988)	Africa	*T. turgidum* L., ssp. *durum* Desf.	49.7 (9)
	Europe	*T. turgidum* L., ssp. *durum* Desf.	48.1 (19)
	North America	*T. turgidum* L., ssp. *durum* Desf.	51.8 (1)
	South America	*T. turgidum* L., ssp. *durum* Desf.	49.0 (2)
**Average**			**48.7 (31)**
Végh *et al.* (2018)	Europe	*T. aestivum* L.	**41.8 (5)**
Rekika *et al.* (1997)	Africa	*T. turgidum* L., ssp. *durum* Desf.	37.0 (1)
	North America	*T. turgidum* L., ssp. *durum* Desf.	35.0 (1)
	Europe	*T. turgidum* L., ssp. *durum* Desf.	37.2 (3)
		*T. turgidum* L., ssp. *diococcoides* Thell.	38.0 (1)
	Europe (wild wheat)	*Aegilops species*	38.2 (5)
**Average**			**37.5 (11)**
Data from nine studies (see Supplementary Table S5)	Asia	*T. aestivum*L.	43.8 (21)
	Africa	*T. aestivum*L.	45.2 (1)
	Australia	*T. aestivum* L. and *T. dicoccum* Schrank	44.5 (79)
	North America		43.9 (32)
**Average**			**44.2 (133)**

^*a*^ The fluorescence temperature response curves used in these studies were similar (ramp rate of 1–1.5 °C min^−1^, in the 20–65 °C range). Values in bold are study averages and those in parentheses indicate the number of genotypes/species used.

## Results

### Diel and phenological variation in *T*_crit_

**Fig. 1.**
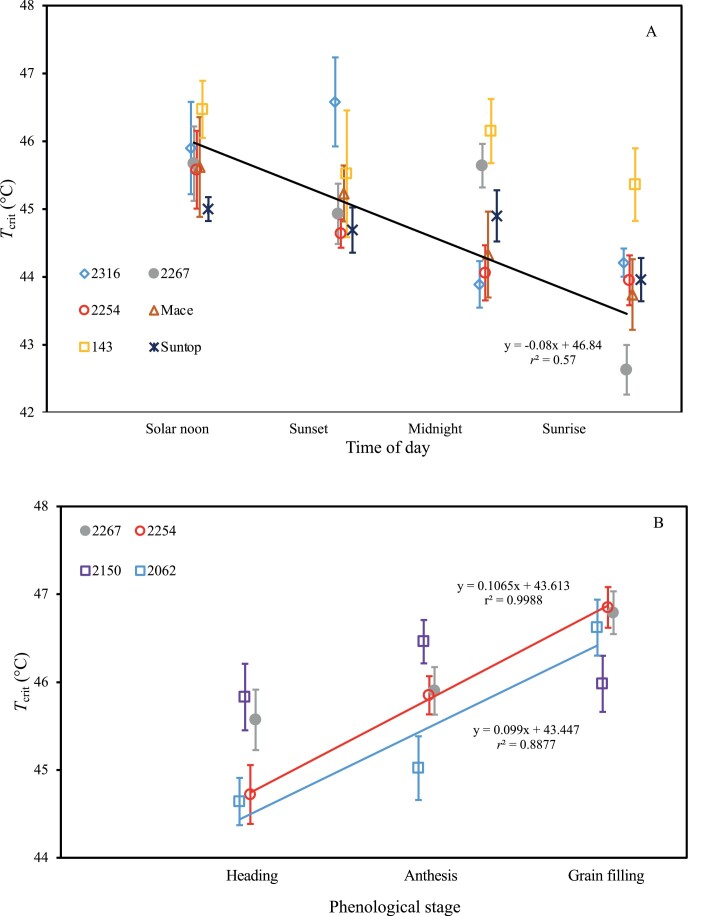
Variation in flag leaf *T*_crit_ (°C) of wheat genotypes over the course of an 18 h period (A), and across three phenological stages (B). Solid lines indicate significant linear trends (*P*<0.05) for all genotypes pooled across time (A) and for genotypes 2254 and 2062 across phenology (B). Plants were grown at field sites in Dingwall, Victoria in 2017 (A), and in Barraport West, Victoria in 2018 (B). Points represent the mean ±SE, *n*=4 for (A) and *n*=8–18 for (B).

Flag leaf chlorophyll fluorescence measurements of 20–24 wheat genotypes (grown at one of three experimental sites in the Australian wheat belt) were used to assess variation in *T*_crit_ across time, phenology, and genotype origin. There was a significant genotype by time of day interaction for *T*_crit_ (*P*=0.042; Supplementary Table S2), highlighting the heterogeneity in this diel variation of *T*_crit_ among our genotypes. In all except genotype 2316, *T*_crit_ tended to be highest at solar noon before then declining through sunset, midnight, and sunrise (Fig. 1A). The slope of these trends was only significant for genotype 2267, with *T*_crit_ declining by 3.1 °C from solar noon to sunrise. In contrast, genotype 143 exhibited the narrowest diel range in *T*_crit_, with difference of 1.1 °C between solar noon and sunrise. Irrespective of genotype, *T*_crit_ at solar noon was significantly higher than at sunrise (*P*<0.001 for time of day). *T*_crit_ also showed a significant genotype by phenology interaction, and highly significant differences for the main effects of genotype as well as phenology (Supplementary Table S2). The interaction effect was largely due to the increasing trend in *T*_crit_ as plants developed from heading to anthesis and grain filling for genotypes 2267, 2254, and 2062, but not for 2150 (Fig. 1B). *T*_crit_ of genotype 2150 rose slightly between heading and anthesis then declined significantly at grain filling relative to anthesis. Genotype 2254 showed the largest increase in *T*_crit_ between heading and anthesis, rising 1.8 °C from 44.4 °C to 46.2 °C.

### Thermal safety margins of Australian wheat

**Fig. 2.**
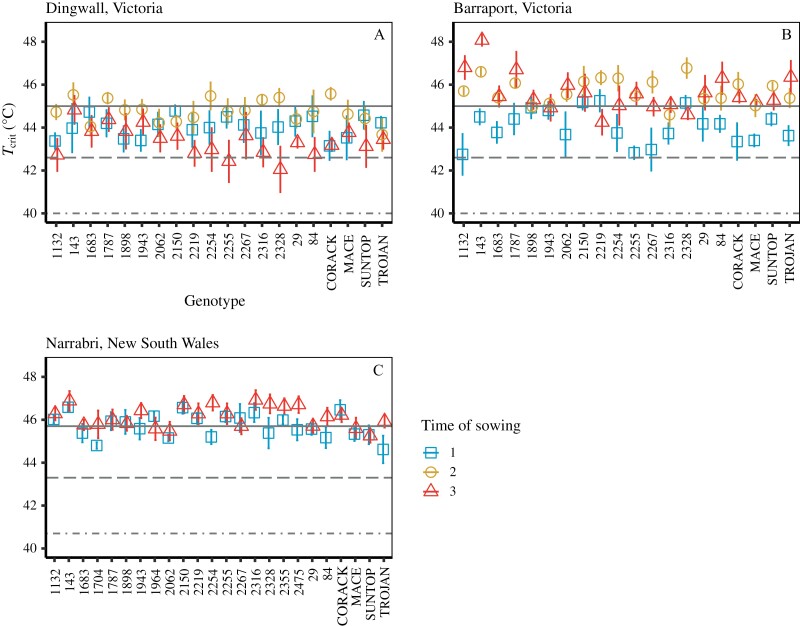
Phenotypic plasticity of leaf *T*_crit_ and thermal safety margins of 20–24 wheat genotypes. The genotypes were sown at either the locally recommended time of year (time of sowing 1; blue squares); 1 month after the recommended time (time of sowing 2; yellow circles); or 2 months after the recommended time (time of sowing 3; red triangles) at three Australian field sites. Delayed times of sowing were used to impose warmer average growth temperatures for plants sown at times of sowing 2 and 3. The field sites were Dingwall (A) and Barraport West (B) Victoria, and Narrabri, New South Wales (C). Twenty genotypes were sown at Dingwall in 2017 and Barraport West in 2018, and the same 20 plus an additional four genotypes were sown at Narrabri in 2019. The dash-dot lines mark the hottest recorded maximum temperature during the typical anthesis month (October) at each field site (40.7 °C for Narrabri, and 40 °C for Dingwall, data from the Australian Bureau of Meteorology; due to the close proximity of Dingwall and Barraport West we used the same climate records for these sites), while the dashed line and the solid line mark the RCP 4.5 IPCC and RCP 8.5 IPCC emission scenarios (+2.6 °C and +5 °C), respectively. The difference between the observed *T*_crit_ and these current and future maximum temperatures is termed the thermal safety margin. Here we assume that leaf temperature is equal to air temperature. Points represent the mean ±SE, minimum *n*=4.

At Dingwall, only the main effect of TOS was significant (Supplementary Table S3). In comparison with TOS 1, *T*_crit_ of TOS2 tended to be higher and TOS 3 tended to be lower (Fig. 2A). At Barraport, *T*_crit_ varied amongst the genotypes over a range of ~2 °C and the effect of TOS on *T*_crit_ depended on the genotype (*P*<0.01 for genotype by TOS interaction). *T*_crit_ increased more in some genotypes (e.g. 1132, 143, and Trojan) under later sowing than others (e.g. 2267 and 29), but also did not change significantly in some (e.g. 1898 and 1943; Fig. 2B). At Narrabri, only the main effects were significant—genotypes varied in their *T*_crit_, and TOS 3 *T*_crit_ was higher than TOS 1 (Fig. 2C; Supplementary Table S3). Across TOS, the genotypes with the lowest and highest mean *T*_crit_ were 1704 (45.3 °C) and 143 (46.7 °C), respectively. Across the 24 genotypes, *T*_crit_ increased by 0.5 °C from TOS 1 (at 45.7 °C) to TOS 3 (at 46.2 °C). An ANOVA run on a linear mixed effects model of the entire field dataset revealed field site to be the largest source of variation in *T*_crit_ of all our independent variables (df = 2, *F*-value = 190.9, *P*<0.001). The overall mean *T*_crit_ at each of the field sites was 45.1 °C at Barraport West, 44.1 °C at Dingwall, and 45.9 °C at Narrabri.

Thermal safety margins were calculated for all field-grown genotypes by quantifying the difference between *T*_crit_ and the maximum air temperature recorded during October. All genotypes demonstrated a higher *T*_crit_ than the historical maximum October air temperatures recorded at each field site (Fig. 2, dash-dot line). Thermal safety margins in the TOS 1 fields ranged from 3.2 °C to 4.8 °C in Dingwall (Fig. 2A), from 2.8 °C to 5.3 °C in Barraport West (Fig. 2B) and from 3.8 °C to 5.8 °C in Narrabri (Fig. 2C). For the later sown crops (i.e. TOS 2 and 3) which experienced warmer growth temperatures, thermal safety margins increased relative to TOS 1 in Dingwall (3.7–5.6 °C for TOS 2), in Barraport West (4.6–6.8 °C for TOS 2, and 4.3–8.1 °C for TOS 3), and in Narrabri (4.5–6.1 °C). The exception to this pattern was TOS 3 at Dingwall where the lower end of the thermal safety margin range declined, resulting in a range of 2.1–4.8 °C. At both Narrabri and Barraport West, mean *T*_crit_ of all genotypes was above the +2.6 °C mark associated with the RCP 4.5 intermediate emission scenario (Fig. 2B, C, dashed line). Most genotypes were also largely clear of the RCP 4.5 mark at Dingwall, except the *T*_crit_ of genotypes 2255 and 2328 sown at TOS 3 which were below this threshold. The +5 °C warming mark associated with the high emission RCP 8.5 scenario was equal to or above the *T*_crit_ of many genotypes at all three field sites, though there was some variation across the locations. At Narrabri, half of the genotypes were below the RCP 8.5 threshold when sown at TOS 1, while this fell to a quarter of genotypes when sown at TOS 3 (Fig. 2C). At TOS 1 in Barraport West, 17 genotypes fell below the RCP 8.5 threshold, with only one and three genotypes below this mark for TOS 2 and 3, respectively (Fig. 2B). At Dingwall, *T*_crit_ of all genotypes sown at TOS 1 and TOS 3 was below the RCP 8.5 threshold, while 14 genotypes at TOS 2 were below this mark (Fig. 2A).

### Genotype origin does not predict variation or acclimation in *T*_crit_

**Fig. 3.**
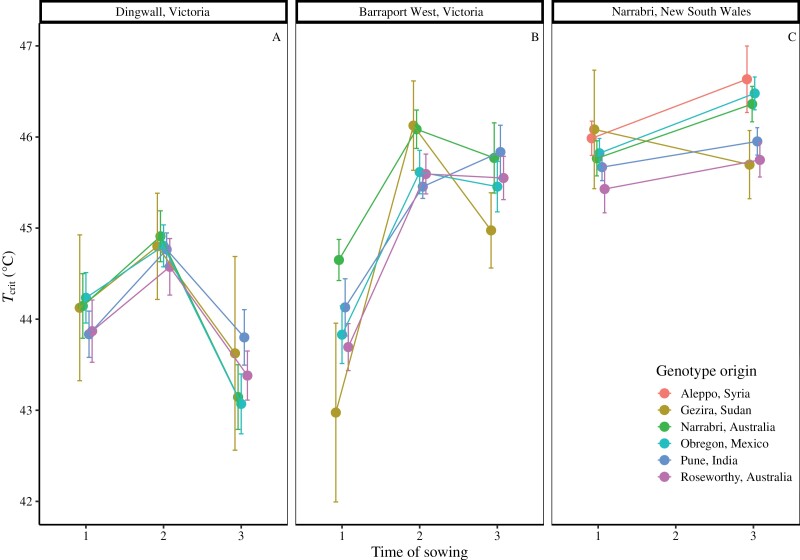
Phenotypic response of wheat flag leaf PSII heat tolerance (*T*_crit_) to time of sowing at three Australian field sites: (A) Dingwall, Victoria; (B) Barraport West, Victoria; and (C) Narrabri, New South Wales. Genotypes are grouped according to the six locations of the breeding programmes where they were developed. Twenty genotypes were grown at Dingwall in 2017 and at Barraport West in 2018, while the same 20 plus an additional four genotypes were grown in Narrabri in 2019. In order to generate increasingly warmer growth temperature regimes, plants were sown at one of three times of sowing: time of sowing 1 (TOS 1) was the locally recommended time of sowing, while TOS 2 and TOS ) were 1 and 2 months after TOS 1, respectively. Points represent the mean ±SE, minimum *n*=4.

The 24 genotypes grown across the three field sites were grouped by the regions from which they originated (Supplementary Table S1; Aleppo, Syria; Gezira, Sudan; Narrabri, Australia; Obregón, Mexico; Pune, India; and Roseworthy, Australia) in order to determine if this explained any of the observed variation in *T*_crit_. Genotype origin had a significant effect on *T*_crit_ at both Barraport West and Narrabri (Supplementary Table S4) At Barraport West, genotypes that originated in Narrabri had the highest *T*_crit_ and Sudan the lowest (Fig. 3B). At Narrabri, the genotype that originated from Syria had the highest *T*_crit_, whereas those from Roseworthy had the lowest. In contrast, genotype origin had no significant effect on *T*_crit_ at Dingwall. TOS had a significant effect on *T*_crit_ at all three sites irrespective of origin. In Dingwall *T*_crit_ was lower for TOS 3 relative to TOS 1 and 2, while in Barraport West *T*_crit_ was lower for TOS 1 than for TOS 2 and 3 (Fig. 3). At the Narrabri site, *T*_crit_ increased from TOS 1 to TOS 3 for all origin groupings (Fig. 3C). No interaction between time of sowing and genotype origin was observed at any field site (Supplementary Table S4).

### Response of *T*_crit_ to short-term exposure to high temperature and upper limit of *T*_crit_ plasticity

**Fig. 4.**
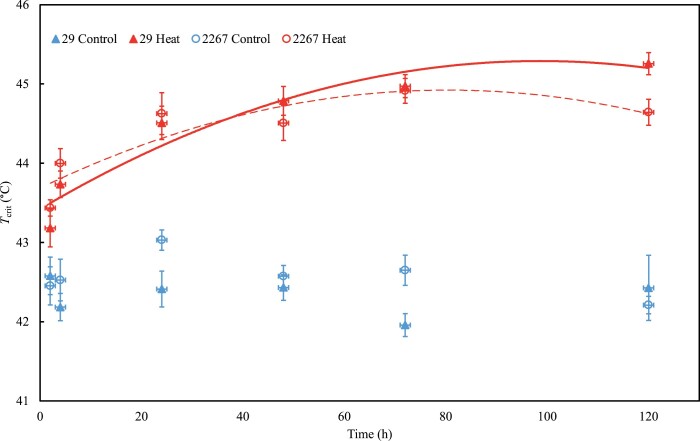
Leaf *T*_crit_ (°C) of two wheat genotypes, 29 (triangles and solid lines) and 2267 (circles and dashed lines), exposed to 24 °C (control; blue shapes and lines) or 36 °C (heat; red shapes and lines) for varying durations (2, 4, 24, 48, 72, or 120 h) in a growth cabinet. Leaf samples for *T*_crit_ were from the third fully extended leaves on the main stem. Equations for the curvilinear relationships between *T*_crit_ at 36 °C (*T*_crit_^36^; °C) and time (*t*; h) under heat for genotype 29 is *T*_crit_^36^=43.42 + 0.038*t*–0.00019*t*^2^ and for genotype 2267 is *T*_crit_^36^=43.69 + 0.031*t*–0.00019*t*^2^. Points represent the mean ±SE, *n*=4.

To characterize the short-term response of *T*_crit_ to high temperature, two genotypes were grown under controlled conditions and measured while at the tillering stage of development. In both genotypes, *T*_crit_ increased significantly following 2 h of heat shock (Fig. 4). As the heat shock progressed, *T*_crit_ increased following a curvilinear pattern which peaked after 3.4 d for genotype 2267 and after 4.2 d for genotype 54. Although the time to reach peak *T*_crit_ during the heat shock differed for the two genotypes, their maximum *T*_crit_ values were similar, being 43.8 °C for genotype 29 and 43.6 °C for genotype 2267 (Fig. 4). *T*_crit_ for both genotypes remained largely constant over the 120 h period for those plants that were maintained at the control day/night temperature regime of 24/12 °C.

### Global variation in wheat *T*_crit_

**Fig. 5.**
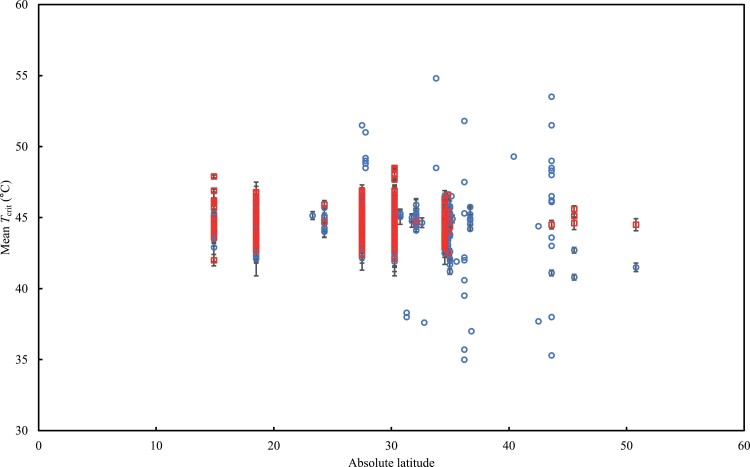
Relationship between *T*_crit_ and the absolute latitude of the climate of origin for wheat genotypes when grown under cool (blue circles) and warm (red squares) conditions. For both conditions, *P*>0.1. Data obtained from 183 wheat genotypes (3223 measurements of leaf *T*_crit_ overall) from experiments in Australia (this study) and the published literature (Havaux *et al.*, 1988; Rekika *et al.*, 1997; Végh *et al.*, 2018). Data points represent the mean *T*_crit_ (±SE where visible) for each genotype.

We combined data from our experiments with previously published data (covering genotypes grown across field and controlled-environment experiments) to examine the degree of variation in *T*_crit_ in wheat genotypes on a global scale based on the latitude of origin as a proxy for climate of origin of their pedigree (Fig. 5). We found three studies (Havaux *et al.*, 1988; Rekika *et al.*, 1997; Végh *et al.*, 2018) that reported wheat leaf *T*_crit_ using similar fluorescence temperature response curves (with ramp rates of 1–1.5 °C min^−1^ between 20 °C and 65 °C) to estimate *T*_crit_. Our final data collation comprised 183 wheat species/varieties (comprising *T. aestivum* L., *T. turgidum* L., ssp. *durum* Desf., *T. turgidum* L., ssp. *diococcoides* Thell., and wild wheat *Aegilops* species) originating from all continents except Antarctica (Table 2). Globally, wheat leaf *T*_crit_ varied by up to 20 °C (35–55 °C) and there were more data for studies under warm conditions for genotypes originating from the lower latitudes than from high latitudes (Fig 5). The larger variation in *T*_crit_ for genotypes originating from the higher latitudes coincided with the cooler growth conditions. Overall, there was less variation in *T*_crit_ under the warm conditions. We found no relationship between mean wheat leaf *T*_crit_ and the absolute latitude of genotype climate of origin (Fig. 5).

## Discussion

In this study, we used a high-throughput technique to record minimal Chl *a* fluorescence and quantified the critical temperature (*T*_crit_) of PSII damage—a measure of leaf photosynthetic heat tolerance—for wheat genotypes grown in multiple field experiments, as well as a controlled-environment experiment. The field experiments demonstrated the extent of variation in *T*_crit_ over the course of a single day, as well as across several crucial stages of phenological development. They also showed that the region of origin of wheat genotypes was unrelated to *T*_crit_ in three representative Australian wheat-growing regions, and that sowing time (and thus, growth temperature) was responsible for significant variation in *T*_crit_. Delayed sowing (i.e. elevated growth temperature) was generally associated with increases in *T*_crit_, resulting in higher thermal safety margins at both field sites. When two genotypes were subjected to a sudden heat shock in a controlled environment, we observed a slight difference between genotypes in the speed with which *T*_crit_ increased. However, both genotypes exhibited a similar peak *T*_crit_ value during this heat shock. Finally, when combining these data with previously published wheat *T*_crit_ data, as well as unpublished data from other experiments conducted in controlled-environment facilities at The Australian National University, we found that the absolute latitude of pedigrees of wheat genotypes were not significantly linked with variation in *T*_crit_ for either cool- or warm-grown plants.

### Temporal fluctuations in wheat *T*_crit_ may be linked to changes in leaf sugar content

Wheat *T*_crit_ varied significantly over the course of a single day, declining by an average of 1.7 °C over the 18 h from solar noon to sunrise (Fig. 1A). This pattern resembles the extent of change in *T*_crit_ in a temperate tree species reported by Hüve *et al.* (2006); specifically, a linear increase over 14 h, from a low point at 05.00 h to a peak at 19.00 h. Taken together, these findings suggest that *T*_crit_ generally increases to a peak during the late afternoon before declining to a minimum between midnight and dawn. Hüve *et al.* (2006) linked this diel variation in *T*_crit_ with daily variation in leaf sugar content, and demonstrated that *T*_crit_ increased when leaves were fed sugar solutions. Further work is needed to determine if the diel variation in *T*_crit_ that we observed in wheat was also influenced by corresponding variation in leaf sugar content.

It is also interesting to compare the extent of variation in *T*_crit_ that was observed over the course of a single day with the extent of variation that was observed across phenology. In the 18 h between solar noon and sunrise. *T*_crit_ declined by 1.7 °C (Fig. 1A), a fluctuation that was similar in size to the 1.5 °C rise in *T*_crit_ that we observed from heading to anthesis (Fig. 1B). This comparison highlights the high level of plasticity in *T*_crit_, and that variation in *T*_crit_ is clearly responsive to factors on both an hours-long time scale (i.e. diurnal fluctuations in leaf sugar content) and a longer term weeks-long scale (as evidenced by changes in *T*_crit_ from heading to anthesis and grain filling). Anthesis is widely considered the stage of phenology at which wheat is most vulnerable to high temperature (Ferris *et al.*, 1998; Thistlethwaite *et al.*, 2020), with this vulnerability largely due to a reduction of sink strength to import and utilize assimilates within the reproductive organs, rather than of assimilate supply from leaf photosynthesis *per se* (Li *et al.*, 2012; Ruan *et al.*, 2012). While this increase could reflect an ongoing rise in heat tolerance coinciding with seasonal warming, there was no significant difference in *T*_crit_ between plants undergoing anthesis versus those at the grain-filling stage, and so anthesis may be the phenological stage at which *T*_crit_ reaches its peak.

### Drivers of variation in wheat *T*_crit_

The field site at which plants were grown was the most significant source of variation in *T*_crit_; the overall average *T*_crit_ at Narrabri was 1.8 °C higher than recorded at Dingwall and 0.8 °C higher than at Barraport West. In addition to environment, genotype had a significant effect on *T*_crit_ at the Barraport West and Narrabri sites. These results suggest that environment, genotype, and most probably the genotype by environment interaction (GxE) may play large roles in determining wheat flag leaf *T*_crit_. Breeding for genotypes with greater photosynthetic heat tolerance (i.e. higher *T*_crit_) may be challenging if variation in *T*_crit_ is also influenced by GxE effects. GxE effects have been reported for other abiotic stress tolerance traits including lodging tolerance in spring wheat (Dreccer *et al.*, 2020), and drought tolerance in maize (Dias *et al.*, 2018).

### Genotypes maintain moderate photosynthetic thermal safety margins

We observed variation in the thermal safety margins of wheat genotypes, predominantly associated with differences between field sites and the effect of sowing time at these sites. The thermal safety margin was 2.1 °C when averaged across all genotypes (Fig. 2). Thermal safety margins in three representative Australian wheat-growing regions were at least 2–4 °C for all genotypes. *T*_crit_ was always several degrees greater than the hottest recorded air temperature during the typical month of anthesis at each site (Narrabri, 40.8 °C; Dingwall/Barraport West 40 °C; denoted by the dot-dash lines in Fig. 2). Under the IPCC’s RCP 4.5 intermediate emission scenario for Eastern Australia by 2090, most genotypes would maintain a positive, yet reduced, thermal safety margin in the studied growing regions. However, under the high emission RCP 8.5 scenario, the thermal safety margins of most genotypes grown at the Dingwall site and a few genotypes at the Barraport West site would be exceeded (Fig. 2A, B). For genotypes grown at the Narrabri site, thermal safety margins under the RCP 8.5 scenario would be drastically reduced and, in some cases, disappear (Fig. 2C). According to our *T*_crit_ observations, only genotypes originating from Obregón and Aleppo would retain positive thermal safety margins under the RCP 8.5 scenario when sown at either optimal or delayed sowing times. The rise in *T*_crit_ with delayed sowing (and thus increased growth temperature) that we observed in the majority of genotypes indicates a widespread capacity for the thermal acclimation of wheat flag leaf *T*_crit_. This suggests that thermal safety margins for wheat photosynthetic heat tolerance could yet increase in response to warming under future climate scenarios. However, given that we also observed an apparent limit to the acclimation of *T*_crit_ following sudden heat shock (Fig. 4), it is possible that daytime maximum temperatures could approach this physiological thermal limit of wheat PSII if the most severe global warming predictions are borne out. A hard limit to the high temperature acclimation of *T*_crit_ could indicate a physiological limitation of PSII, or a temperature that represents the absolute maximum tolerance. Given that the considerable thermal plasticity of PSII electron transport has been closely linked with improving photosynthetic heat tolerance more generally (Yamasaki *et al.*, 2002), the prospect of air temperatures approaching the physiological threshold of PSII high temperature acclimation is concerning.

Thus far, in assessing thermal safety margins, we have assumed parity between air and leaf temperatures; however, wheat leaf/canopy temperature can differ substantially from air temperature. Balota *et al.* (2007) reported canopy temperatures ranging from 3 °C below noon air temperatures to 10 °C above noon air temperatures in dryland wheat, and from 3 °C below noon air temperatures to 5.7 °C above noon air temperatures in irrigated wheat. Similarly, canopy temperatures of Australian wheat have also been recorded exceeding afternoon air temperature by 0.3–2.3 °C (Rattey *et al.*, 2011) and 3–5 °C (Rebetzke *et al.*, 2013). These examples, along with other previous instances (Rashid *et al.*, 1999; Thapa *et al.*, 2018), highlight the significant genotypic variation in canopy cooling and thus the potential for achieving gains in performance under high temperature by exploiting this variation. While greater levels of canopy cooling could increase thermal safety margins by limiting leaf temperature, achieving gains in wheat *T*_crit_ could also provide an avenue to maintaining positive thermal safety margins by increasing the threshold to PSII damage. Enhancing thermal safety margins by increasing *T*_crit_ could be particularly important in water-limited environments considering that heatwaves are frequently accompanied by drought, which increases stomatal closure and limits transpirational cooling, resulting in increased leaf temperature (Aspinwall *et al.*, 2019).

### Thermal environment of growth site may be more influential than genotype origin in determining variation in wheat flag leaf *T*_crit_

Considering the potential benefits to wheat heat tolerance and performance under high temperature that could arise from achieving increases in *T*_crit_, as well as the extent of variation that we observed in *T*_crit_ among 24 genotypes at three field sites, it would be beneficial to identify characteristics that predict high *T*_crit_ in wheat genotypes. Thus, we analysed whether the distinct regions from which our genotypes originated could reliably predict variation in *T*_crit_. Previous studies of (mostly) woody, non-crop species found that *T*_crit_ was correlated with climate of origin (O’Sullivan *et al.*, 2017; Zhu *et al.*, 2018). In a similar vein, we found evidence of genotype region of origin significantly affecting *T*_crit_ at two of our field sites (Fig. 3A, C). One consistency at both of these sites was that genotypes originating from Roseworthy, Australia generally exhibited the lowest or second lowest mean *T*_crit_ values. In contrast, the genotype from Aleppo exhibited the highest *T*_crit_ at the Narrabri site (Fig. 3C), while at the Barraport West site the genotypes originating from Narrabri had the highest mean *T*_crit_ across all sowing times (Fig. 3A). However, it seems unlikely that the effect of genotype region of origin is the result of differences in temperature at these locations: for instance, the average daily maximum April temperature in Aleppo, Syria is 23 °C (NOAA), while the average daily October maximum in Roseworthy, Australia is 23.8 °C (Australian Bureau of Meteorology). Therefore, the differences associated with genotype origin in the current study are likely to be related to a more complex combination of environmental differences between locations (e.g. rainfall, temperature, soil quality, and agricultural practices). Differences in the aims and methods of breeding programmes at various locations could also explain variation in *T*_crit_ associated with genotype origin. We also note that our experiments did not include genotypes that were developed in cooler environments, such as wheat-growing regions in Europe or Northern America, and so further work may be required to capture the full extent of global variation in wheat *T*_crit_.

### *T*_crit_ increases within hours of heat shock, and peaks after 3–4 d

We observed widespread evidence of wheat *T*_crit_ plasticity following exposure to high temperature, including elevated growth temperature in the field (via delayed sowing, Figs 2, 3) and sudden heat shock under controlled conditions (Fig. 4). We also saw clear genotypic variation in the plasticity of *T*_crit_ across these experiments. In some genotypes, *T*_crit_ rose by upwards of 4 °C when sowing time was delayed by 2 months (Fig. 2B), while in others *T*_crit_ showed no change or even declined by up to 1.2 °C from TOS 1 to TOS 3 (Fig. 2A). Similarly, following a heat shock imposed under controlled conditions, we observed a difference between two genotypes in the speed at which *T*_crit_ increased despite the two genotypes eventually reaching a similar peak *T*_crit_ (Fig. 4). Genotypic variation is thus evident not only in wheat flag leaf *T*_crit_ under common non-stressful temperatures, but also in the extent of *T*_crit_ plasticity in response to sudden heat shock. Increases in *T*_crit_ with warming have been reported previously (O’Sullivan *et al.*, 2017; Zhu *et al.*, 2018), and these are considered examples of high temperature acclimation. That we observed similar patterns in wheat *T*_crit_, as well as genotypic variation in this acclimation, suggests that the capacity to increase PSII heat tolerance could be a trait worth targeting for the development of wheat genotypes with greater heat tolerance. However, further work is needed to first investigate whether such acclimation is associated with enhanced performance under high temperature in the field.

One aspect of the current study that may aid such future efforts is the development of high-throughput minimal Chl *a* fluorescence assays that can be used for large-scale screening of wheat PSII heat tolerance. When combined with other burgeoning high-throughput techniques for measuring photosynthetic characteristics (Sharma *et al.*, 2012; Silva-Pérez *et al.*, 2018; Fu *et al.*, 2019; McAusland *et al.*, 2019; Arnold *et al.*, 2021), it is becoming increasingly achievable to efficiently measure a range of traits that provide insight into the photosynthetic thermal tolerance of entire plots in crop breeding trials.

### Genotypic variation in wheat leaf *T*_crit_ is not consistent with latitudinal trends in general plant heat tolerance

In contrast to previous results that observed a decrease in PSII heat tolerance (measured as *T*_crit_) as latitude moved further from the equator (O’Sullivan *et al.*, 2017; Lancaster and Humphreys, 2020), we found no relationship between wheat leaf *T*_crit_ and the latitude of genotype climate of origin, irrespective of thermal acclimation (Fig. 5). This discrepancy could be related to differences between cultivated and wild species: the studies of O’Sullivan *et al.* (2017) and Lancaster and Humphreys (2020) demonstrated a relationship between heat tolerance and latitude based almost entirely on records of different wild species. In contrast, our study focuses solely on one domesticated species. Wheat is known as a crop with a particularly narrow genetic background (Tanksley and McCouch, 1997), but we observed a large range of *T*_crit_ in wheat here (up to 20 °C) which compares with the ~30 °C global range reported across 218 plant species spanning seven biomes reported by O’Sullivan *et al.* (2017). This large range of wheat leaf *T*_crit_ can be exploited to improve heat tolerance in modern crop varieties, as has been done recently in successful efforts to improve wheat drought tolerance (Reynolds *et al.*, 2015). Still, wheat is cultivated in a wide range of ecological and climatic conditions, covering >220 Mha, including areas where it is exposed to high temperature stress. As such, we predicted that the rise in *T*_crit_ that we observed with elevated growth temperature in our experimental dataset (Figs 1–4) would also be apparent in the meta-analysis. However, there was no evidence of any thermal acclimation response of *T*_crit_ in this larger dataset. This could partly be due to the diversity of experimental methods used to generate the data in Fig. 5, as well as variation in the duration and intensity of elevated growth temperature treatments. Given that the plant thermal tolerance field uses a large and diverse range of experimental designs and assays (Geange *et al.*, 2021), the results of our systematic review of wheat *T*_crit_ could be further evidence of a need to better standardize the approaches used to measure and describe photosynthetic heat tolerance.

### Conclusion

Wheat leaf *T*_crit_ varied dynamically with changes in growth conditions, notably increasing in response to short- and long-term high temperatures, and exhibiting an upper ceiling in acclimating to heatwaves. There was also evidence of developmental, diel, and genotypic variation in *T*_crit_, as well as a strong genotype-by-environment interaction. Interestingly, global wheat leaf *T*_crit_ which spanned up to 20 °C was unrelated to genotype climate of origin and latitude, unlike reported associations with global interspecies variation in leaf *T*_crit_ of 171 plant species (~30 °C). However, the observed genotypic variation and plasticity of wheat *T*_crit_, combined with the recent development of a high-throughput technique for measuring *T*_crit_ (Arnold *et al* 2021), indicate that this trait would be useful for high-throughput screening, understanding photosynthetic heat tolerance, and the development of heat-tolerant wheat.

## Supplementary data

The following supplementary data are available at *JXB* online.

Table S1. Wheat pedigree information for genotypes grown in the three field experiments and one controlled-environment experiment described in this study.

Table S2. ANOVA of factors influencing wheat *T*_crit_ at two Australian field sites.

Table S3. ANOVA of effect of time of sowing and genotype on wheat *T*_crit_ at three Australian field sites.

Table S4. ANOVA of effect of time of sowing and genotype origin on wheat *T*_crit_ at three Australian field sites.

Table S5. Mean wheat leaf *T*_crit_ from nine studies conducted under controlled-environment and field conditions in Australia. 

erac039_suppl_Supplementary-Materials

## Data Availability

Data supporting the findings of this study are available from the corresponding author upon request.

## References

[CIT0001] Addo-Bediako A , Chown SL, Gaston KJ. 2000. Thermal tolerance, climatic variability and latitude. Proceedings of the Royal Society B: Biological Sciences 267, 739–745. 10.1098/rspb.2000.1065 PMC1690610 10819141

[CIT0002] Araújo MB , Ferri-Yáñez F, Bozinovic F, Marquet PA, Valladares F, Chown SL. 2013. Heat freezes niche evolution. Ecology Letters 16, 1206–1219. 23869696 10.1111/ele.12155

[CIT0003] Armond PA , Schreiber U, Björkman O. 1978. Photosynthetic acclimation to temperature in the desert shrub, *Larrea divaricata*. Plant Physiology 61, 411–415. 16660304 10.1104/pp.61.3.411 PMC1091879

[CIT0004] Arnold PA , Briceño VF, Gowland KM, Catling AA, Bravo LA, Nicotra AB. 2021. A high-throughput method for measuring critical thermal limits of leaves by chlorophyll imaging fluorescence. Functional Plant Biology 48, 634–646. 33663678 10.1071/FP20344

[CIT0005] Aspinwall MJ , Pfautsch S, Tjoelker MG, et al. 2019. Range size and growth temperature influence Eucalyptus species responses to an experimental heatwave. Global Change Biology 25, 1665–1684. 30746837 10.1111/gcb.14590

[CIT0006] Asseng S , Ewert F, Martre P, et al. 2015. Rising temperatures reduce global wheat production. Nature Climate Change 5, 143–147.

[CIT0007] Balota M , Payne WA, Evett SR, Lazar MD. 2007. Canopy temperature depression sampling to assess grain yield and genotypic differentiation in winter wheat. Crop Science 47, 1518–1529.

[CIT0008] Berry J , Bjorkman O. 1980. Photosynthetic response and adaptation to temperature in higher plants. Annual Review of Plant Physiology 31, 491–543.

[CIT0009] Brestic M , Zivcak M, Kalaji HM, Carpentier R, Allakhverdiev SI. 2012. Photosystem II thermostability *in situ*: environmentally induced acclimation and genotype-specific reactions in *Triticum aestivum* L. Plant Physiology and Biochemistry 57, 93–105. 22698752 10.1016/j.plaphy.2012.05.012

[CIT0010] Climate Change Australia. 2021. Climate change in Australia: climate information, projections, tools and data. Available at: https://www.climatechangeinaustralia.gov.au/en/

[CIT0011] Coast O , Posch BC, Bramley H, Gaju O, Richards RA, Lu M, Ruan Y, Trethowan R, Atkin OK. 2021. Acclimation of leaf photosynthesis and respiration to warming in field-grown wheat. Plant, Cell & Environment 44, 2331–2346. 10.1111/pce.13971 33283881

[CIT0012] Coast O , Shah S, Ivakov A, et al. 2019. Predicting dark respiration rates of wheat leaves from hyperspectral reflectance. Plant, Cell & Environment 42, 2133–2150. 10.1111/pce.13544 30835839

[CIT0013] Cossani CM , Reynolds MP. 2012. Physiological traits for improving heat tolerance in wheat. Plant Physiology 160, 1710–1718. 23054564 10.1104/pp.112.207753 PMC3510104

[CIT0014] Deutsch CA , Tewksbury JJ, Huey RB, Sheldon KS, Ghalambor CK, Haak DC, Martin PR. 2008. Impacts of climate warming on terrestrial ectotherms across latitude. Proceedings of the National Academy of Sciences, USA 105, 6668–6672. 10.1073/pnas.0709472105 PMC2373333 18458348

[CIT0015] Dias KODG , Gezan SA, Guimarães CT, et al. 2018. Estimating genotype × environment interaction for and genetic correlations among drought tolerance traits in maize via factor analytic multiplicative mixed models. Crop Science 58, 72–83.

[CIT0016] Drake JE , Tjoelker MG, Vårhammar A, et al. 2018. Trees tolerate an extreme heatwave via sustained transpirational cooling and increased leaf thermal tolerance. Global Change Biology 24, 2390–2402. 29316093 10.1111/gcb.14037

[CIT0017] Dreccer MF , Condon AG, Macdonald B, et al. 2020. Genotypic variation for lodging tolerance in spring wheat: wider and deeper root plates, a feature of low lodging, high yielding germplasm. Field Crops Research 258, 107942.

[CIT0018] Ferguson JN , McAusland L, Smith KE, Price AH, Wilson ZA, Murchie EH. 2020. Rapid temperature responses of photosystem II efficiency forecast genotypic variation in rice vegetative heat tolerance. The Plant Journal 104, 839–855. 32777163 10.1111/tpj.14956

[CIT0019] Ferris R , Ellis RHR, Wheeler TR, Hadley P. 1998. Effect of high temperature stress at anthesis on grain yield and biomass of field-grown crops of wheat. Annals of Botany 82, 631–639.

[CIT0020] Figueroa FL , Conde-Álvarez R, Gomez I. 2003. Relations between electron transport rates determined by pulse amplitude modulated chlorophyll fluorescence and oxygen evolution in macroalgae under different light conditions. Photosynthesis Research 75, 259–275. 16228606 10.1023/A:1023936313544

[CIT0021] Fu P , Meacham-Hensold K, Guan K, Bernacchi CJ. 2019. Hyperspectral leaf reflectance as proxy for photosynthetic capacities: an ensemble approach based on multiple machine learning algorithms. Frontiers in Plant Science 10, 730. 31214235 10.3389/fpls.2019.00730 PMC6556518

[CIT0022] Gabriel W , Lynch M. 1992. The selective advantage of reaction norms for environmental tolerance. Journal of Evolutionary Biology 5, 41–59.

[CIT0023] Geange SR , Arnold PA, Catling AA, et al. 2021. The thermal tolerance of photosynthetic tissues: a global systematic review and agenda for future research. New Phytologist 229, 2497–2513. 33124040 10.1111/nph.17052

[CIT0024] Havaux M , Ernez M, Lannoye R. 1988. Correlation between heat tolerance and drought tolerance in cereals demonstrated by rapid chlorophyll fluorescence tests. Journal of Plant Physiology 133, 555–560.

[CIT0025] Hochman Z , Gobbett DL, Horan H. 2017. Climate trends account for stalled wheat yields in Australia since 1990. Global Change Biology 23, 2071–2081. 28117534 10.1111/gcb.13604

[CIT0026] Hoffmann AA , Chown SL, Clusella-Trullas S. 2013. Upper thermal limits in terrestrial ectotherms: how constrained are they? Functional Ecology 27, 934–949.

[CIT0027] Hüve K , Bichele I, Rasulov B, Niinemets U. 2011. When it is too hot for photosynthesis: heat-induced instability of photosynthesis in relation to respiratory burst, cell permeability changes and H_2_O_2_ formation. Plant, Cell & Environment 34, 113–126. 10.1111/j.1365-3040.2010.02229.x 21029116

[CIT0028] Hüve K , Bichele I, Tobias M, Niinemets U. 2006. Heat sensitivity of photosynthetic electron transport varies during the day due to changes in sugars and osmotic potential. Plant, Cell & Environment 29, 212–228. 10.1111/j.1365-3040.2005.01414.x 17080637

[CIT0029] Iqbal M , Raja NI, Yasmeen F, Hussain M, Ejaz M, Shah MA. 2017. Impacts of heat stress on wheat: a critical review. Advances in Crop Science and Technology 5, 1–9.

[CIT0030] Kuznetsova A , Brockhoff PB, Christensen RHB. 2017. lmerTest package: tests in linear mixed effects models. Journal of Statistical Software 82, doi :10.18637/jss.v082.i13.

[CIT0031] Lancaster LT , Humphreys AM. 2020. Global variation in the thermal tolerances of plants. Proceedings of the National Academy of Sciences, USA 117, 13580–13587. 10.1073/pnas.1918162117 PMC7306813 32482870

[CIT0032] Lenth R. 2020. Estimated marginal means, aka least-squares means. https://github.com/rvlenth/emmeans

[CIT0033] Leon-Garcia IV , Lasso E. 2019. High heat tolerance in plants from the Andean highlands: implications for paramos in a warmer world. PLoS One 14, e022. 10.1371/journal.pone.0224218 PMC6834248 31693675

[CIT0034] Leung C , Rescan M, Grulois D, Chevin L. 2020. Reduced phenotypic plasticity evolves in less predictable environments. Ecology Letters 23, 1664–1672. 32869431 10.1111/ele.13598 PMC7754491

[CIT0035] Li ZM , Palmer P, Martin M, Wang R, Rainsford F, Jin Y, Patrick JW, Yang YJ, Ruan Y-L. 2012. High invertase activity in tomato reproductive organs correlates with enhanced sucrose import into, and heat tolerance of, young fruit. Journal of Experimental Botany 63, 1155–1166. 22105847 10.1093/jxb/err329 PMC3276082

[CIT0036] Liu B , Martre P, Ewert F, et al. 2019. Global wheat production with 1.5 and 2.0°C above pre-industrial warming. Global Change Biology 25, 1428–1444. 10.1111/gcb.14542 30536680

[CIT0037] McAusland L , Atkinson JA, Lawson T, Murchie EH. 2019. High throughput procedure utilising chlorophyll fluorescence imaging to phenotype dynamic photosynthesis and photoprotection in leaves under controlled gaseous conditions. Plant Methods 15, 109. 31548849 10.1186/s13007-019-0485-x PMC6749646

[CIT0038] Melcarek PK , Brown GN. 1979. Chlorophyll fluorescence monitoring of freezing point exotherms in leaves. Cryobiology 16, 69–73. 436440 10.1016/0011-2240(79)90012-9

[CIT0039] Muggeo VMR. 2008. Segmented: an R package to fit regression models with broken-line relationships. R News 8, 20–25.

[CIT0040] Neuner G , Pramsohler M. 2006. Freezing and high temperature thresholds of photosystem II compared to ice nucleation, frost and heat damage in evergreen subalpine plants. Physiologia Plantarum 126, 196–204.

[CIT0041] Ortiz-Bobea A , Wang H, Carrillo CM, Ault TR. 2019. Unpacking the climatic drivers of US agricultural yields. Environmental Research Letters 14, 064003.

[CIT0042] O’Sullivan OS , Heskel MA, Reich PB, et al. 2017. Thermal limits of leaf metabolism across biomes. Global Change Biology 23, 209–223. 27562605 10.1111/gcb.13477

[CIT0043] Perez TM , Feeley KJ. 2020. Photosynthetic heat tolerances and extreme leaf temperatures. Functional Ecology 34, 2236–2245.

[CIT0044] Perkins-Kirkpatrick SE , Lewis SC. 2020. Increasing trends in regional heatwaves. Nature Communications 11, 3357. 10.1038/s41467-020-16970-7 PMC7334217 32620857

[CIT0045] Raison JK , Roberts JKM, Berry JA. 1982. Correlations between the thermal stability of chloroplast (thylakoid) membranes and the composition and fluidity of their polar lipids upon acclimation of the higher plant, *Nerium oleander*, to growth temperature. Biochimica et Biophysica Acta 688, 218–228.

[CIT0046] Rashid FAA , Scafaro AP, Asao S, Fenske R, Dewar RC, Masle J, Taylor NL, Atkin OK. 2020. Diel- and temperature-driven variation of leaf dark respiration rates and metabolite levels in rice. New Phytologist 228, 56–69. 32415853 10.1111/nph.16661

[CIT0047] Rashid A , Stark JC, Tanveer A, Mustafa T. 1999. Use of canopy temperature measurements as a screening tool for drought tool for drought tolerance in spring wheat. Journal of Agronomy and Crop Science 182, 231–238.

[CIT0048] Rattey AR , Shorter R, Chapman SC. 2011. Evaluation of CIMMYT conventional and synthetic spring wheat germplasm in rainfed sub-tropical environments. II. Grain yield components and physiological traits. Field Crops Research 124, 195–204.

[CIT0049] R Core Team. 2018. R: a language and environment for statistical computing. Vienna, Austria: R Foundation for Statistical Computing.

[CIT0050] Rebetzke GJ , Rattey AR, Farquhar GD, Richards RA, Condon AG. 2013. Genomic regions for canopy temperature and their genetic association with stomatal conductance and grain yield in wheat. Functional Plant Biology 40, 14–33. 10.1071/FP12184 32481083

[CIT0051] Rekika D , Monneveux E, Havaux M. 1997. The in vivo tolerance of photosynthetic membranes to high and low temperatures in cultivated and wild wheats of the *Triticum* and *Aegilops* genera. Journal of Plant Physiology 150, 734–738.

[CIT0052] Reynolds M , Tattaris M, Cossani CM, Ellis M, Yamaguchi-Shinozaki K, Saint Pierre C. 2015. Exploring genetic resources to increase adaptation of wheat to climate change. In: Ogihara Y, Takumi S, Handa H, eds. Advances in wheat genetics: from genome to field. Tokyo: Springer Japan, 355–368.

[CIT0053] Ruan Y-L , Patrick JW, Bouzayen M, Osorio S, Fernie AR. 2012. Molecular regulation of seed and fruit set. Trends in Plant Science 17, 656–665. 22776090 10.1016/j.tplants.2012.06.005

[CIT0054] Sastry A , Barua D. 2017. Leaf thermotolerance in tropical trees from a seasonally dry climate varies along the slow–fast resource acquisition spectrum. Scientific Reports 7, 11246. 28900253 10.1038/s41598-017-11343-5 PMC5595873

[CIT0055] Scafaro AP , Atkin OK. 2016. The impact of heat stress on the proteome of crop species. In: Salekdeh G, ed. Agricultural Proteomics Volume 2. Cham: Springer International Publishing, 155–175.

[CIT0056] Scafaro AP , Negrini ACA, O’Leary BM, et al. 2017. The combination of gas-phase fluorophore technology and automation to enable high-throughput analysis of plant respiration. Plant Methods 13, 1–13. 28344635 10.1186/s13007-017-0169-3 PMC5361846

[CIT0057] Scheiner SM. 1993. Genetics and evolution of phenotypic plasticity. Annual Review of Ecology and Systematics 24, 35–68.

[CIT0058] Schreiber U , Berry JA. 1977. Heat-induced changes of chlorophyll fluorescence in intact leaves correlated with damage of the photosynthetic apparatus. Planta 136, 233–238. 24420396 10.1007/BF00385990

[CIT0059] Schreiber U , Colbow K, Vidaver W. 1975. Temperature-jump chlorophyll fluorescence induction in plants. Zeitschrift für Naturforschung C 30, 689–690. 10.1515/znc-1975-9-1026 130002

[CIT0060] Sharkey TD. 2005. Effects of moderate heat stress on photosynthesis: importance of thylakoid reactions, rubisco deactivation, reactive oxygen species, and thermotolerance provided by isoprene. Plant, Cell & Environment 28, 269–277.

[CIT0061] Sharma DK , Andersen SB, Ottosen CO, Rosenqvist E. 2012. Phenotyping of wheat cultivars for heat tolerance using chlorophyll a fluorescence. Functional Plant Biology 39, 936–947. 32480843 10.1071/FP12100

[CIT0062] Silva-Pérez V , Molero G, Serbin SP, Condon AG, Reynolds MP, Furbank RT, Evans JR. 2018. Hyperspectral reflectance as a tool to measure biochemical and physiological traits in wheat. Journal of Experimental Botany 69, 483–496. 29309611 10.1093/jxb/erx421 PMC5853784

[CIT0063] Slot M , Cala D, Aranda J, Virgo A, Michaletz ST, Winter K. 2021. Leaf heat tolerance of 147 tropical forest species varies with elevation and leaf functional traits, but not with phylogeny. Plant, Cell & Environment 44, 2414–2427. 10.1111/pce.14060 33817813

[CIT0064] Slot M , Krause GH, Krause B, Hernández GG, Winter K. 2019. Photosynthetic heat tolerance of shade and sun leaves of three tropical tree species. Photosynthesis Research 141, 119–130. 30054784 10.1007/s11120-018-0563-3

[CIT0065] Steer BT. 1973. Diurnal variations in photosynthetic products and nitrogen metabolism in expanding leaves. Plant Physiology 51, 744–748. 16658402 10.1104/pp.51.4.744 PMC366338

[CIT0066] Sunday JM , Bates AE, Dulvy NK. 2011. Global analysis of thermal tolerance and latitude in ectotherms. Proceedings of the Royal Society B: Biological Sciences 278, 1823–1830. 10.1098/rspb.2010.1295 PMC3097822 21106582

[CIT0067] Sunday JM , Bates AE, Kearney MR, Colwell RK, Dulvy NK, Longino JT, Huey RB. 2014. Thermal-safety margins and the necessity of thermoregulatory behavior across latitude and elevation. Proceedings of the National Academy of Sciences, USA 111, 5610–5615. 10.1073/pnas.1316145111 PMC3992687 24616528

[CIT0068] Tack J , Barkley A, Nalley LL. 2015. Effect of warming temperatures on US wheat yields. Proceedings of the National Academy of Sciences, USA 112, 6931–6936. 10.1073/pnas.1415181112 PMC4460489 25964323

[CIT0069] Tanksley SD , McCouch SR. 1997. Seed banks and molecular maps: unlocking genetic potential from the wild. Science 277, 1063–1066. 9262467 10.1126/science.277.5329.1063

[CIT0070] Thapa S , Jessup KE, Pradhan GP, Rudd JC, Liu S, Mahan JR, Devkota RN, Baker JA, Xue Q. 2018. Canopy temperature depression at grain filling correlates to winter wheat yield in the U.S. Southern High Plains. Field Crops Research 217, 11–19.

[CIT0071] Thistlethwaite RJ , Tan DKY, Bokshi AI, Ullah S, Trethowan RM. 2020. A phenotyping strategy for evaluating the high-temperature tolerance of wheat. Field Crops Research 255, 107905.

[CIT0072] Végh B , Marček T, Karsai I, Janda T, Darkó E. 2018. Heat acclimation of photosynthesis in wheat genotypes of different origin. South African Journal of Botany 117, 184–192.

[CIT0073] Way DA , Yamori W. 2014. Thermal acclimation of photosynthesis: on the importance of adjusting our definitions and accounting for thermal acclimation of respiration. Photosynthesis Research 119, 89–100. 23812760 10.1007/s11120-013-9873-7

[CIT0074] Weng JH , Lai MF. 2005. Estimating heat tolerance among plant species by two chlorophyll fluorescence parameters. Photosynthetica 43, 439–444.

[CIT0075] Yamasaki T , Yamakawa T, Yamane Y, Koike H, Satoh K, Katoh S. 2002. Temperature acclimation of photosynthesis and related changes in photosystem II electron transport in winter wheat. Plant Physiology 128, 1087–1097. 11891263 10.1104/pp.010919 PMC152220

[CIT0076] Yamashita A , Nijo N, Pospíšil P, Morita N, Takenaka D, Aminaka R, Yamamoto Y, Yamamoto Y. 2008. Quality control of photosystem II: reactive oxygen species are responsible for the damage to photosystem II under moderate heat stress. Journal of Biological Chemistry 283, 28380–28391. 18664569 10.1074/jbc.M710465200 PMC2661399

[CIT0077] Zadoks JC , Chang TT, Konzak CF. 1974. A decimal code for the growth stages of cereals. Weed Research 14, 415–421.

[CIT0078] Zhao C , Liu B, Piao S, et al. 2017. Temperature increase reduces global yields of major crops in four independent estimates. Proceedings of the National Academy of Sciences, USA 114, 9326–9331. 10.1073/pnas.1701762114 PMC5584412 28811375

[CIT0079] Zhu L , Bloomfield KJ, Hocart CH, Egerton JJG, O’Sullivan OS, Penillard A, Weerasinghe LK, Atkin OK. 2018. Plasticity of photosynthetic heat tolerance in plants adapted to thermally contrasting biomes. Plant, Cell & Environment 41, 1251–1262. 10.1111/pce.13133 29314047

