# Supplementary material for: Wheat photosystem II heat tolerance responds dynamically to short- and long-term warming
Source: J Exp Bot. 2022 May 23;73(10):3268–82. doi: 10.1093/jxb/erac039 (PMC9127437; doi:10.1093/jxb/erac039)
Supplement: erac039_suppl_Supplementary-Materials [file erac039_suppl_supplementary-materials.pdf]

Supplementary materials

Table S1. Pedigree information for wheat genotypes grown for the three field studies and one controlled environment study described in the materials and methods.

Reference no.	Pedigree	Note	Group, geographical origin
**Field studies**			
84	Sokoll/2/Sokoll/ 35888 M 500132	Backcross of a hexaploid synthetic derived wheat to a heat tolerant tetraploid *T. dicoccum* and a hexaploid type selected	Narrabri, Australia
1132	PBW550//C80.1/*2Batavia	Cross of heat tolerant Indian cultivar with rust resistant sources	Pune, India
1683	PBW343+L24+LR28/Lang	Same as above	Pune, India
1787	DBW16/Sunstate	Same as above	Pune, India
1898	DBW16/Annuello	Same as above	Pune, India
1943	DBW16/Gladius	Same as above	Pune, India
2062	ISR 812.8/Carinya (1, sister line)	Heat tolerant Mexican hexaploid landrace cross to Australian cultivar	Obregon, Mexico
2150	ISR 812.8/Carinya (2, sister line)	Same as above	Obregon, Mexico
2219	Ventura/Ido 637//Ventura	Low phytate mutant crossed to Australian cultivar - pre-screened for heat tolerance	Narrabri, Australia
2254	D67.2/P66.270//AE.Squarrosa (320)/3/Cunningham/4/Vorb	Heat tolerant in Mexico (Ciudad Obregon) and Narrabri, Australia. Origin CGIAR	Obregon, Mexico
2255	SLVS/Attila//WBLL1*2/3/Gondo/CBRD	Same as above	Obregon, Mexico
2328	Sokoll/2/Sokoll/35888 M 500132	Backcross of a hexaploid synthetic wheat to a heat tolerant tetraploid *T. dicoccum* and a hexaploid type selected	Narrabri, Australia
Corack		Commercial Australian cultivar, released in 2012	Roseworthy, Australia
Suntop		Same as above	Roseworthy, Australia
Trojan		Commercial Australian cultivar, released in 2013	Roseworthy, Australia
Mace	Wyalkatchem/Stylet//Wyalkatchem	Commercial Australian cultivar, released in 2008	Roseworthy, Australia
2475	Attila/3*BCN//Bav92/3/Tilhi/5/Bav92/3/PRL/Sara//TSI/Vee#5/4/Croc_1/Ae.Squarrosa (224)//2*Opata	Heat tolerant in Mexico (Ciudad Obregon) and Narrabri. Origin CGIAR	Obregon, Mexico
2355	Seri 82/Shuha's'//CM85295-0101TOPY-2M-0Y-0M-3Y-0M-0AP	Same as above	Aleppo, Syria
1964	DBW14/C80.1/2*SR2 Batavia	Cross of heat tolerant Indian cultivar with rust resistant sources	Pune, India
1704	PBW343+L24+LR28/Lang	Same as above	Pune, India

29	Berkut/2/Berkut/35883 M500110	Backcross of a hexaploid wheat to a heat tolerant tetraploid *T. dicoccum* and a hexaploid type selected	Narrabri, Australia
143	Waxwing*2/Kiritati /3/Waxwing*2/Kiritati /2/ 35888 M 500132	Same as above	Narrabri, Australia
2316	RAC 1192/4/2*Attila/3/Weaver*2/TSC//Weaver	Heat tolerant hexaploid; good performance in Mexico (Ciudad Obregon) and Narrabri, Australia. Origin CGIAR	Obregon, Mexico
**Field studies and controlled environment study**			
2267	Hubara-8///Mon's'/Ald's'//Bow's'	Heat tolerant hexaploid; good performance in Sudan and Narrabri. Origin CGIAR	Gezira, Sudan

Table S2. Analysis of variance of factors influencing wheat T_{crit} at two Australian field sites

	**Genotype**		**Time of day**		**Genotype x Time of day**	
	d.f.	*F* value	d.f.	*F* value	d.f.	*F* value
Dingwall, Victoria	5	3.7 **	3	15.8 ***	15	2 *
	**Genotype**		**Phenological stage**		**Genotype x Phenological stage**	
	d.f.	*F* value	d.f.	*F* value	d.f.	*F* value
Barraport West, Victoria	3	5.0**	2	14.9 ***	6	2.2 *

$^{*}P < 0.05$; $^{**}P < 0.01$; $^{***}P < 0.001$. Six out of the 20 genotypes sown at Dingwall in 2017 were sampled every six hours to measure diel variation in T_{crit} over the course of a day. Four of the 20 genotypes sown at Barraport West in 2018 were sampled from all three time of sowing plots to measure variation in wheat T_{crit} at varying phenological stages.

Table S3. Analysis of variance of effect of time of sowing and genotype on wheat T_{crit} at three Australian field sites

	**Time of sowing**		**Genotype**		**Time of sowing x Genotype**	
	d.f.	*F* value	d.f.	*F* value	d.f.	*F* value
Dingwall, Victoria	2	23.1 ***	19	0.5 ns	38	0.9 ns
Barraport West, Victoria	2	62.1 ***	19	2.1 **	38	2 **
Narrabri, New South Wales	1	13.7 ***	23	2.3 ***	23	1 ns

*$P < 0.05$; **$P < 0.01$; ***$P < 0.001$; ns = not significant. The same 20 genotypes that were sown in Dingwall and Barraport West were also sown at Narrabri in 2019, along with an additional four genotypes.

Table S4. Analysis of variance of effect of time of sowing and genotype origin on wheat T_{crit} at three Australian field sites

	**Time of sowing**		**Genotype origin**		**Time of sowing x Genotype origin**	
	d.f.	*F* value	d.f.	*F* value	d.f.	*F* value
Dingwall, Victoria	2	15.2 ***	4	0.2 ^{ns}	8	0.7 ^{ns}
Barraport West, Victoria	2	42.2 ***	4	2.5 *	8	1 ^{ns}
Narrabri, New South Wales	1	4.5 *	5	2.5 *	5	0.7 ^{ns}

$^{*}P < 0.05$; $^{**}P < 0.01$; $^{***}P < 0.001$; ^{ns} = not significant. The same 20 genotypes that were sown in Dingwall and Barraport West were also sown at Narrabri in 2019, along with an additional four genotypes.
